# Statistical evaluation of synchronous spike patterns extracted by frequent item set mining

**DOI:** 10.3389/fncom.2013.00132

**Published:** 2013-10-23

**Authors:** Emiliano Torre, David Picado-Muiño, Michael Denker, Christian Borgelt, Sonja Grün

**Affiliations:** ^1^Institute of Neuroscience and Medicine (INM-6) and Institute for Advanced Simulation (IAS-6), Jülich Research Centre and JARAJülich, Germany; ^2^European Centre for Soft ComputingMieres, Spain; ^3^Theoretical Systems Neurobiology, RWTH Aachen UniversityAachen, Germany

**Keywords:** higher-order correlations, neuronal cell assemblies, spike patterns, spike synchrony, multiple testing, data mining

## Abstract

We recently proposed frequent itemset mining (FIM) as a method to perform an optimized search for patterns of synchronous spikes (*item sets)* in massively parallel spike trains. This search outputs the occurrence count (*support*) of individual patterns that are not trivially explained by the counts of any superset (*closed frequent item sets*). The number of patterns found by FIM makes direct statistical tests infeasible due to severe multiple testing. To overcome this issue, we proposed to test the significance not of individual patterns, but instead of their signatures, defined as the pairs of pattern size *z* and support *c*. Here, we derive in detail a statistical test for the significance of the signatures under the null hypothesis of full independence (*pattern spectrum filtering*, PSF) by means of surrogate data. As a result, injected spike patterns that mimic assembly activity are well detected, yielding a low false negative rate. However, this approach is prone to additionally classify patterns resulting from chance overlap of real assembly activity and background spiking as significant. These patterns represent false positives with respect to the null hypothesis of having one assembly of given signature embedded in otherwise independent spiking activity. We propose the additional method of *pattern set reduction* (PSR) to remove these false positives by conditional filtering. By employing stochastic simulations of parallel spike trains with correlated activity in form of injected spike synchrony in subsets of the neurons, we demonstrate for a range of parameter settings that the analysis scheme composed of FIM, PSF and PSR allows to reliably detect active assemblies in massively parallel spike trains.

## 1. Introduction

The cortex is comprised of a highly interconnected network of neurons and thus one may speculate that information processing in the brain may only be understood on the basis of the concerted activity of the neuronal population. Hebb (1949) suggested that neurons coordinate their activities by organizing in functional groups, termed cell assemblies. Synchronous spike input to receiving neurons is known to be more effective in generating output spikes (Abeles, 1982; König et al., 1996), which leads to the hypothesis that temporal coordination of spiking activity or correlational processing is the defining expression of an active cell assembly (Singer et al., 1997; Harris, 2005). As excitatory postsynaptic potentials are small in amplitude compared to the gap between the resting potential and the neuronal firing threshold, it is expected that a cell assembly is composed of many neurons firing in a correlated fashion. This observation is the basis for the assumption that higher-order synchronous spiking activity serves as a signature expression of an active assembly (Riehle et al., 1997; Berger et al., 2010; Staude et al., 2010b; Shimazaki et al., 2012).

In order to observe and detect such signatures in the brain, the spiking activities of many neurons must be recorded simultaneously. Fortunately, in recent years considerable progress has been made in the development of multi-electrode recording techniques [e.g., Nicolelis, 1998; Buzsaki, 2004; Hatsopoulos et al., 2007; Riehle et al., 2013], which enable to record the activity of hundred(s) of neurons. Such massively parallel spike train data pose statistical challenges due to the inherent complexity of the required multivariate approaches. Most notably, increasing the number of observed neurons leads to a combinatorial explosion of the number of potential spike patterns that need to be detected and tested. Based on pairwise correlation analyses only, the existence and functional relevance of neuronal correlations could be demonstrated in various cortical systems and behavioral paradigms [e.g., Gerstein and Aertsen, 1985; Riehle et al., 1997; Kohn and Smith, 2005; Berger et al., 2007; Fujisawa et al., 2008; Feldt et al., 2009; Humphries, 2011; Masud and Borisyuk, 2011]. Nevertheless, a correlation analysis considering the complete set of simultaneously recorded spike trains is required to uncover also higher-order correlations among neurons. In recent years several such approaches were developed, each of which focuses on different aspects: (i) methods to determine the presence of higher-order spike correlations with a minimum order without explicitly identifying the participating neurons [e.g., Louis et al., 2010a; Staude et al., 2010a,b]; (ii) methods that test whether individual neurons participate in synchronous spiking activity without identifying the groups of correlated neurons [e.g., Berger et al., 2010]; (iii) methods that test for the presence of correlation as predicted by a specific correlation model such as a synfire chain (Abeles, 1991), that is, spatio-temporal spike patterns or propagation of synchronous spiking activity [e.g., Abeles and Gerstein, 1988; Schrader et al., 2008; Gerstein et al., 2012; Gansel and Singer, 2012]; (iv) methods that directly identify the members of cell assemblies on the basis of the patterns of synchronous spiking activity [e.g., Gerstein et al., 1978; Pipa et al., 2008; Feldt et al., 2009; Gansel and Singer, 2012; Shimazaki et al., 2012; Picado-Muiño et al., 2013].

In Picado-Muiño et al. (2013) we presented the basic approach and relevant statistics to employ frequent item set mining (FIM) to identify significant patterns of spike synchrony in massively parallel spike trains. FIM enables fast and efficient counting of synchronous spike patterns by pruning the tree of all possible patterns. To address the problem of multiple testing, statistics are not computed for individual patterns, but on the pattern spectrum that collects the number of observed patterns based on their signature. A signature is defined as the pair (*z, c*) of pattern size *z* (i.e., number of participating neurons) and *support c* (i.e., number of pattern occurrences). In *pattern spectrum filtering* (PSF) those identified sets of neurons for which patterns with the same signature (*z, c*) occur also in appropriate surrogate data are then marked as chance patterns and discarded.

Here, we extend the approach of Picado-Muiño et al. (2013) in three ways that will enable the application of the method to biological data. First, we refine the statistical test employed in pattern spectrum filtering for reporting significant patterns of a given signature (Section 2). Then, we introduce a subsequent analysis step, termed *pattern set reduction* (PSR), to additionally filter out those patterns that are detected as significant, but are compositions of chance spikes or patterns and the actual cell assembly pattern (Section 3). Finally, we report on the performance of our method related to features describing the data (e.g., coincidence rate, assembly pattern size, firing rate heterogeneity or non-stationarity) and analysis parameters (Section 4). The discussion (Section 5) includes a step-by-step instruction on how to utilize the proposed method in the context of massively parallel spike trains obtained from electrophysiological recordings.

## 2. Spike pattern detection and statistical testing

In this section we introduce our approach to detect frequent synchronous spike patterns in massively parallel spike trains (MPST). We first briefly review frequent item set mining (FIM) and related terminology and definitions as proposed in Picado-Muiño et al. (2013) as a tool to efficiently detect and count synchronous spike patterns in MPST. Then we derive a modified version of the FIM-based statistics proposed in Picado-Muiño et al. (2013) for assessing pattern significance.

### 2.1. Frequent itemset mining

**Figure 1:**
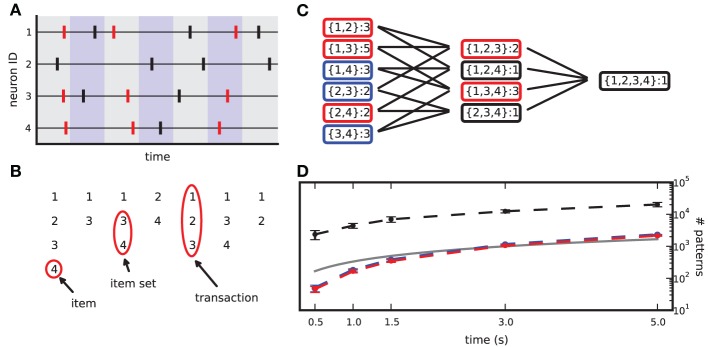
**From spike data to closed frequent itemsets. (A)** Sketch of a raster plot of 4 neurons firing in parallel. Shaded colors separate adjacent bins. Red spikes mark the occurrences of the synchronous pattern composed of neurons 1,3,4. **(B)** Transaction list derived from the spike data in **(A)** after binning. **(C)** List of item sets obtained from **(B)**, together with their occurrence counts. Black boxes mark non-frequent item sets (support set to 2), blue boxes mark non-closed frequent item sets, red boxes mark CFISs. **(D)** Average number of item sets (dashed black line), frequent item sets (dashed blue line) and CFISs (dashed red line) obtained from 100 simulations of 100 parallel independent spike trains with a firing rate of 20 Hz, as a function of the simulation time. Other parameters are bin width *w* = 3 ms and minimum pattern size *z*_0_ = 2. Bars mark ±1 std. dev. The solid line indicates the number of time bins (and thus transactions) as a function of the simulation time.

Given *N* parallel spike trains with neuron ids 1,2, …,*N*, observed in the time window [0, *T*), we partition [0, *T*) into *b* exclusive bins {b_*i*_}^*b*^_*i* = 1_ of identical width *w* = *T/b* (typically chosen as a few ms): *b*_*i*_ = [(*i* − 1) · *w, i* · *w*). If one or more spikes of one neuron fall into a bin, we consider the bin occupied and reduce the entry to 1 (*clipping*), so that each time bin contains at most one spike per neuron. Spikes from different neurons falling into the same time bin are defined as *synchronous* (see Figure 1A). Borrowing terminology from FIM, we define each neuron id as an *item*, the set *T*_*i*_ of all items spiking in *b*_*i*_ as the *i*-th *transaction* in the binned data, and {*T*_*i*_}^*b*^_*i* = 1_ as the *transaction list*. Given a *minimum pattern size z*_0_, each set of *z* ≥ *z*_0_ items in *T*_*i*_ constitutes a *pattern of synchronous spikes*, or *item set* (see Figure 1B). Here we set *z*_0_ to 2. Due to clipping, each item set occurs at most once per transaction. The number of occurrences of an item set in the transaction list is the *support* of that item set.

A transaction that contains *K* items yields 2^*K*^ − *K* − 1 different (but possibly overlapping) item sets of size *z* ≥ 2, that is, all 2^*K*^ possible subsets without the empty set and the *K* singletons. The total number of different item sets in a transaction list can thus largely exceed the number of transactions (i.e., time bins). This number grows with the duration of the data set (see Figure 1D) and with the number of parallel spike trains (not shown).

In order to limit the data to potentially interesting and non-trivial item sets, we select only item sets whose support *c* is larger than or equal to a *minimum support c*_0_ (*c*_0_ ≥ 1) as introduced by Picado-Muiño et al. (2013). Here we set *c*_0_ to 2. An item set whose support equals or exceeds the minimum support is called *frequent item set*. For *c*_0_ > 1, frequent item sets are usually a small fraction of all item sets (Figure 1D, compare black dashed line to blue dashed line). Furthermore, we discard any frequent item set occurring as many times as any of its supersets. These patterns are trivially explained by the occurrences of their supersets, which are more significant due to the larger number of neurons involved. Non-trivial frequent item sets are called *closed frequent item sets* (CFISs; see Figure 1C). Discarding non-closed frequent item sets does not yield any loss of information. Indeed, the set 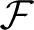 of all frequent item sets can be reconstructed from the set 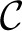 of CFISs by

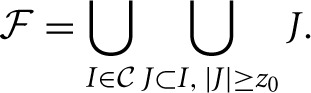



The support *s*(*I*) of a non-closed frequent item set *I* ∈ 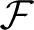 can be computed as *s*(*I*) = max_*J* ∈ *C, J* ⊃ *I*_*s*(*J*).

If *A* and *B* are two CFISs such that *B* ⊊ *A*, and *c*_*A*_, *c*_*B*_ their respective supports, it follows from the definition of CFISs that *c*_*B*_ > *c*_*A*_ (*a priori* property). We refer to the (non-empty) set *A*\*B* as the *excess items* of *A* with respect to *B*, and to the difference *c*_*B*_ − *c*_*A*_ as the *excess occurrences* of *B* with respect to *A*.

Following Picado-Muiño et al. (2013), we make use of frequent itemset mining [FIM; for a review, see Goethals (2010), Borgelt (2012)] to extract CFISs and their support from an MPST transaction list. FIM performs a non-redundant search for spike patterns, starting from those of size *z*_0_ and then moving on to supersets of increasing size. Starting at lowest-size patterns, the search is organized in a search tree in layers of increasing pattern size. A branch connects two patterns if one is a subset of the other. Each pattern is visited at most once. FIM exploits the *apriori* property to stop the search at infrequent patterns, as no supersets of an infrequent item set can be frequent. The output of FIM is a list of all CFISs with their support (Figure 1C).

### 2.2. Pattern spectrum filtering

Direct statistical tests of all individual patterns occurring in MPST are not suitable, as they cause a severe multiple testing problem yielding large occurrences of false positives (FPs), or enhanced levels of false negatives (FNs) after statistical corrections. Therefore Picado-Muiño et al. (2013) proposed to pool CFISs according to their size *z* (number of neurons involved) and their support *c* (number of occurrences) in a two-dimensional histogram (*pattern spectrum*) and to evaluate patterns of the same signature (*z, c*) for significance by a Monte-Carlo approach using surrogate data. Here we present a refinement of this original approach, named *pattern spectrum filtering* (PSF), that bases the test for a specific signature (*z, c*) also on patterns of higher size and support than specified by the signature.

**Figure 2:**
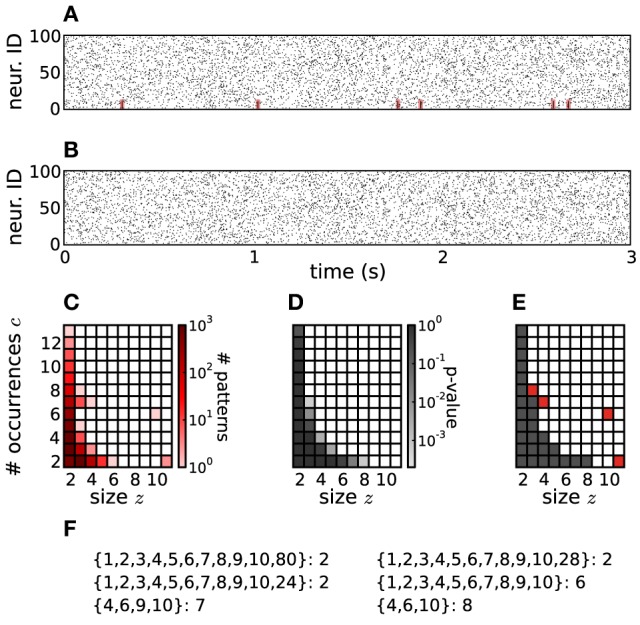
**PSF on artificial data. (A)** Raster plot of 100 parallel simulated spike trains consisting of independent Poisson activity plus 6 injections of one pattern of synchronous spikes (highlighted in red) from neurons 1 to 10, occurring at random times (see Section 4 for details). The total firing rate of each neuron is 20 Hz, the simulation time is 3 s. **(B)** Same as in **(A)**, but without injection of synchronous patterns. The spike trains are therefore completely independent. **(C)** Pattern spectrum of CFISs extracted from the data in **(A)** by FIM (*z*_0_ = 2, *c*_0_ = 2, *w* = 5 ms). Counts are color-coded (logarithmic scale). **(D)** *P*-value spectrum drawn from 5000 surrogate, independent data sets of the type shown in **(B)**. *P*-values are color-coded (logarithmic scale). **(E)** Significance spectrum (overall significance α = 0.01, Bonferroni-corrected for *m* = 50 tests yielding α_*_ = 2·10^−4^). Gray squares indicate signatures that are not significant, white squares mark potentially significant signatures. Red squares mark significant signatures of the pattern spectrum shown in **(C)**, i.e., which fall into white squares of the significance spectrum. **(F)** List of patterns detected by PSF. Besides the injected pattern *A* = {1, 2, 3, 4, 5, 6, 7, 8, 9, 10}, PSF also classifies additional patterns as significant, all being subsets or supersets of *A*.

In order to implement the null hypothesis 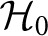 of independent spiking, and to approximate the *p*-values of the signatures (*z, c*), from the original data (Figure 2A) we repeatedly generate surrogate data (Figure 2B), collect from each one its CFISs through FIM as done for the original data, and compute the corresponding surrogate pattern spectrum (Figure 2C). The surrogates are generated from the original data by intentionally destroying correlations while keeping other features, such as firing rates, intact [e.g., by spike randomization or spike dithering, Louis et al. (2010b)].

Let ≽ be the partial ordering on the real plane, that is, (x_*_, y_*_) ≽ (*x, y*) if x_*_ ≥ *x* and y_*_ ≥ *y*, where ≻ holds if at least one inequality is strict. From each surrogate pattern spectrum we compute a binary spectrum which takes value 1 at each signature (*z, c*) such that at least one signature (z_*_, c_*_) ≽ (*z, c*) is occupied, and value 0 otherwise [in contrast to Picado-Muiño et al. (2013) where only the occupation of signature (*z, c*) is checked]. Formally, we define the *signature operator* sgt(·) such that, given a CFIS *A* with size *z*_*A*_ = |*A*| and occurrence count *c*_*A*_, sgt(*A*) := (*z*_*A*_, *c*_*A*_). For each list 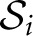 of CFISs from one surrogate data set, let $\hat{P}$_*i*_ be the *binary pattern spectrum*, defined for each *z, c* ≥ 2 by:

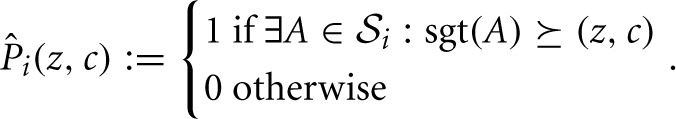



Averaging the binary spectra at each signature, we get the *p-value spectrum $\hat{P}$:*

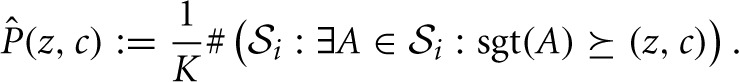



$\hat{P}$(*z, c*) yields an estimate of the probability to observe (one or more) patterns with signature (z_*_, c_*_) ≽ (*z, c*) under 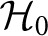 (see Figure 2D).

We then classify any signature (*z, c*) whose *p*-value is lower than the significance level α_*_ as significant. Given the desired overall significance level α for PSF, we derive α_*_ from α by Bonferroni correction for the number *m* of tests, i.e., the number of signatures in the data to test for: α_*_ = α/m. Any signature (*z, c*) for which $\hat{P}$(*z, c*) < α_*_ is classified as significant. Formally, we introduce the *significance spectrum Ŝ* defined at each *(*z, c*)* by

$$\hat{S}(z,c):=\{\begin{array}{ll} 1 & \text{if}(z,c)\text{issignificant}\\ 0 & \text{otherwise} \end{array}\;.$$

In Figure 2E *Ŝ*(*z, c*) = 1 is marked in white, *Ŝ*(*z, c*) = 0 in gray. The border between the two is the *detection border*, on the left of which signatures in the original data are classified as not significant and rejected. Signatures to its right (*Ŝ*(*z, c*) = 1) are considered as significant (marked in red in Figure 2E). The corresponding patterns and their supports are listed in Figure 2F.

## 3. Pattern set reduction

**Figure 3:**
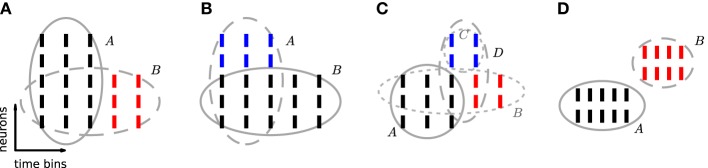
**Excess occurrences and excess items.** Sketch of the possible relationship between a reference pattern and patterns sharing neuron identities and/or time occurrences with it. In each panel, ticks represent individual spikes. Rows correspond to neurons and columns to transactions, i.e., time bins. Spikes forming a pattern are grouped by an ellipse. The reference pattern of each panel is shown by black ticks and is indicated by a solid ellipse. **(A)** *B* is a subset of *A* with excess occurrences (red). **(B)** *A* is a superset of *B* with excess items (blue). **(C) *B*** is a subset of *A* with excess occurrences (red). Neurons in *C* (blue) additionally fire synchronously to *A* and to excess occurrences of *B*. Thus pattern *D* = *B* ∪ *C* forms a CFIS, which partially overlaps with *A*. **(D)** Patterns *A* and *B* are disjoint: they are composed of different neuron identities and occur at different time bins.

PSF tests the significance of patterns under the null hypothesis 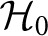 of fully uncorrelated spike trains. However, PSF might fail in rejecting patterns that result from combinations of chance spikes or chance patterns with the assembly pattern (see list of detected patterns in Figure 2F besides the injected one). These patterns are a specific kind of false positive, not resulting from merely independent data. They may be subsets or supersets of the assembly pattern, or patterns that partially overlap with it (Figures 3A–C). In this section we define the type of FPs that may occur, investigate why PSF is prone to return such FPs, and propose an additional statistical analysis, termed *pattern set reduction* (PSR), to remove them.

### 3.1. Types of FPs

#### 3.1.1. Chance subsets

If a CFIS *A* repeats *c*_*A*_ times and a subset *B* of *A* (with |*B*| ≥ *z*_0_) has *c* additional chance occurrences, *B* represents a CFIS repeating *c*_*B*_ = *c*_*A*_ + *c* total times. We call *B* a *chance subset* of *A*, having *c* excess occurrences (Figure 3A). PSF is designed to test the significance of signature (|*B*|, *c*_*B*_) under 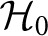 (complete independence), thus disregarding the fact that *c*_*A*_ occurrences are due to pattern *A*. As a result it classifies *B* as a significant pattern, thus yielding an FP outcome. This is illustrated in Figure 2F, where e.g., pattern {4, 6, 10} occurs twice by chance plus 6 times as a subset of pattern {1, 2, 3, 4, 5, 6, 7, 8, 9, 10}. The corresponding signature (3,8) is significant compared to the surrogates (Figure 2E), so that PSF does not reject it.

#### 3.1.2. Chance supersets

If a CFIS *B* occurs *c*_*B*_ times and another set *C* of neurons fires by chance synchronously with *B* in *c* of those *c*_*B*_ transactions (with *c* ≥ *c*_0_), then the pattern *A* = *B* ∪ *C* represents a CFIS repeating *c*_*A*_ = *c* times. We call *A* a *chance superset* of *B*, with |*C*| excess neurons (Figure 3B). PSF tests the significance of signature (|*A*|, *c*_*A*_) under 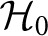, disregarding the fact that |*B*| of the |*A*| neurons of *A* are due to the presence of pattern *B*. The test is therefore prone to classify *A* as significant. This is the case for patterns {1, 2, …, 10, 80}, {1, 2, …, 10, 28} and {1, 2, …, 10, 24} in Figure 2F, each of which occurs twice as a superset of {1, 2, …, 10}. The corresponding signature (11,2) is significant compared to the surrogates (Figure 2E), so that PSF classifies these patterns as significant.

#### 3.1.3. Chance overlapping sets

The simultaneous presence of excess items and excess occurrences can yield yet another type of FP outcome, namely patterns that overlap with the actual assembly. Given an assembly *A*, assume that a subset *B* of *A* has additional chance occurrences. If an additional set *C* of neurons disjoint from *A* fires synchronously to *A and* to an excess occurrence of *B* for a total of *c* ≥ *c*_0_ chance times, then the set *D* = *B* ∪ *C* represents a CFIS which partially overlaps with *A* (Figure 3C). PSF is prone to classify *D* as significant.

#### 3.1.4. Disjoint patterns

Two patterns which do not have items in common are *disjoint* (Figure 3D). In contrast to the previous classes of chance patterns, the presence of an active assembly does not enhance chance patterns disjoint from it. PSF therefore correctly estimates their significance and manages to filter out almost all of them, as shown in 4.

### 3.2. PSR statistics

Let 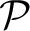 be the class of CFISs reported as significant by PSF. Given a pair (*A, B*) ∈ 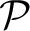 × 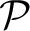 such that *B* ⊂ *A* (therefore *c*_*B*_ > *c*_*A*_ by definition of CFIS, and |*B*| < |*A*|), we propose statistical tests to assess the conditional significance of either *A given B* (*A*|*B*) or *B given A* (*B*|*A*), i.e., of one pattern given that the other represents an assembly pattern. These tests can be applied, using different strategies, to the class of all such (*A, B*) pairs, reducing 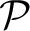 to a subclass 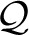 of patterns which are mutually significant given each other.

#### 3.2.1. Subset filtering

This procedure aims at rejecting FPs that are chance subsets of other CFISs. For each pair (*A, B*) ∈ 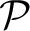 × 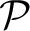 such that *B* ⊂ *A* (so that *c*_*B*_ > *c*_*A*_), *B* has *c*_*B*_ − *c*_*A*_ excess occurrences with respect to *A*. Subset filtering tests *B*|*A*, i.e., the null hypothesis 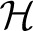
^*B*|*A*^_0_ that *B* is a chance subset of the actual assembly *A*, by assessing the significance of the excess occurrences of *B*. Equivalently, 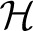
^*B*|*A*^_0_ states that the pattern *B*′ defined by the same items as *B* but its excess occurrences only (red spikes in Figure 3A) is a chance pattern. If 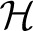
^*B*|*A*^_0_ is rejected, *B* is kept and *A* discarded, otherwise *A* is kept and *B* discarded. Thus, the procedure keeps either *A* or *B* and discards the other (*exclusive*). We present two alternatives to test 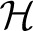
^*B*|*A*^_0_.

***3.2.1.1. Exact test***. This test computes the *p*-value of the signature (|*B*|, *c*_*B*_ − *c*_*A*_) of *B*′. If *c*_*B*_ − *c*_*A*_ < *c*_0_, *B* is classified as a chance subset of *A*. Otherwise, let *T*′_*A*_ be the transaction list obtained from *T* by discarding the transactions where *A* occurred, and keeping in the remaining transactions only the items composing *A*. All the excess occurrences of subsets of *A* must be contained in *T*′_*A*_. *B*′ itself is a CFIS in this transaction list: it is an item set because |*B*′| = |*B*| ≥ *z*_0_, it is frequent because *c*_*B*_ − *c*_*A*_ ≥ *c*_0_, it is closed because otherwise *B* itself would be non-closed. To test the significance of *B*′, one can therefore run FIM and PSF on surrogates of *T*′_*A*_ to estimate the significance of its signature (|*B*|, *c*_*B*_ − *c*_*A*_). If (|*B*|, *c*_*B*_ − *c*_*A*_) is significant, *B*′ is significant in *T*′_*A*_ and *B* is classified as significant in *T* (given *A*). Otherwise, *B* is classified as non-significant.

***3.2.1.2. Approximate test***. This test approximates the *p*-value of the signature (|*B*|, *c*_*B*_ − *c*_*A*_) in *T*′_*A*_ by the *p*-value of the signature (|*B*|, *c*_*B*_ − *c*_*A*_ + *h*), *h* ≥ 1, in *T*, already obtained when performing PSF. In contrast to *T*′_*A*_, *T* is composed of more neurons than those which can actually form chance subsets of *A* (because it does not contain the items of *A* only), and more transactions than those where such subsets could actually display excess occurrences (because it also contains the transaction where *A* is already present). Therefore, the *p*-value of (|*B*|, *c*_*B*_ − *c*_*A*_) would be underestimated if computed over *T* instead of *T*′_*A*_. Parameter *h* heuristically corrects for this by substituting it with the *p*-value of a signature with the same size but higher support. The lower *h*, the higher the probability to reject *B*. If *h* ≥ *c*_*A*_, then (|*B*|, *c*_*B*_ − *c*_*A*_ + *h*) ≽ (|*B*|, *c*_*B*_) and *B* is necessarily reported as significant. This test avoids to run FIM and PSF on *T*′_*A*_ and is therefore computationally more efficient.

#### 3.2.2. Superset filtering

This procedure aims at rejecting FPs that are chance supersets of other CFISs. For each pair (*A, B*) ∈ 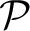 × 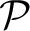 such that *B* ⊂ *A* (so that |*B*| < |*A*|), *A* has |*A*| − |*B*| excess items with respect to *B*. Subset filtering tests *A*|*B*, i.e., the null hypothesis 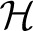
^*A*|*B*^_0_ that *A* is a chance superset of the actual assembly *B*, by assessing the significance of the excess items of *A*. Equivalently, 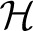
^*A*|*B*^_0_ states that the pattern *A*′ defined by the same transactions as *A* but containing its excess items only (blue spikes in Figure 3B), is a chance pattern. If 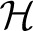
^*A*|*B*^_0_ is rejected, *A* is kept and *B* discarded from 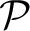, otherwise *B* is kept and *A* discarded from 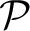. Thus, the procedure keeps either *A* or *B* and discards the other (*exclusive*). We present two alternatives to test 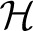
^*A*|*B*^_0_.

***3.2.2.1. Exact test***. This test computes the significance of the signature (|*A*| − |*B*|, *c*_*A*_) of *A*′. If |*A*| − |*B*| < *z*_0_, *A* is classified as a chance superset of *B*. Otherwise, let *T*_*B*_′ be the transaction list obtained from *T* by keeping only the transaction where *B* occurred, and discarding from them the items constituting *B*. All groups of excess items of *B* (i.e., neurons that fire synchronously to *B*) must be contained in *T*_*B*_′. *A*′ itself is a CFIS of this transaction list: it is an item set because |*A*′| = |*A*| − |*B*| ≥ *z*_0_, it is frequent because *c*_*A*_ ≥ *c*_0_, it is closed because otherwise *A* itself would be non-closed. To test the significance of *A*′, one can therefore run FIM and PSF on surrogates of *T*_*B*_′ to estimate the *p*-value of its signature (|*A*| − |*B*|, *c*_*A*_). If (|*A*| − |*B*|, *c*_*A*_) is significant, *A*′ is significant in *T*_*B*_′ and *A* is classified as significant in *T* (given *B*). Otherwise, *A* is classified as non-significant.

***3.2.2.2. Approximate test***. This test approximates the *p*-value of the signature of *A*′ in *T*_*B*_′ by the *p*-value of signature (|*A*| − |*B*| + *k, c*_*A*_), *k* ≥ 1, in *T*, already obtained when performing PSF. In contrast to *T*_*B*_′, *T* is composed of more neurons than those that can actually form excess items of *B* (because it contains the items of *B*, too), and more transactions than those where supersets of *B* could actually occur (because it contains also transactions where *B* does not occur). Therefore, the *p*-value of (|*A*| − |*B*|, *c*_*A*_) would be underestimated if computed over *T* instead of *T*_*B*_′. Parameter *k* heuristically corrects for this by substituting it with the *p*-value of a signature with the same support but higher size. The lower *k*, the higher the probability to reject *A*. Note that if *k* ≥ |*B*| then (|*A*| − |*B*| + *k, c*_*A*_) ≽ (|*A*|, *c*_*A*_) and *A* is necessarily reported as significant. This test allows to avoid running FIM and PSF on *T*_*B*_′ for each *B*.

#### 3.2.3. Covered-spikes criterion

This simple selection strategy consists of taking all pairs (*A, B*) ∈ 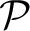 × 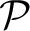 for which *B* ⊂ *A*, and keeping for each pair the pattern covering the largest number of spikes, while rejecting the other. Specifically, the criterion prefers *A* to *B* if *z*_*A*_ · *c*_*A*_ ≥ *z*_*B*_ · *c*_*B*_, *B* to *A* otherwise. It does not involve significance tests, but is based on the observation that, given the probability *p* for a neuron to spike in a time bin, the probability for *z* neurons to fire synchronously in a bin is approximately *p*^*z*^, so that the probability that this pattern occurrs *c* times is binomially distributed and approximately proportional to *p*^*z* · *c*^. The larger the *z* · *c* score, the less likely a pattern of such size and support. This matches the finding that the detection border separating non-significant signatures (marked gray in Figure 2E) from significant ones (marked white in Figure 2E) in the significance spectrum exhibits a hyperbolic shape. The criterion thus keeps the less likely of the two patterns.

A variant consists in keeping the pattern with the largest (*z* − 1) · *c* score. This choice is motivated by the observation that a pattern of size *z* and support *c* can be seen as *z* − 1 spike trains which synchronize their spikes to another train *c* times. Thus, (*z* − 1) · *c* spikes are coincident to spikes in another spike train. Keeping the pattern with the largest (*z* − 1) · *c* score amounts to keeping the pattern which covers more coincident spikes. Geometrically, penalizing the pattern size corrects for the fact that the hyperbolic shape of the detection border in Figure 2E is elongated toward the pattern support (*y*-axis) rather than being equilateral.

#### 3.2.4. Combined filtering

Subset filtering, superset filtering and covered-spikes criterion can be combined into a filtering procedure which tests for both excess coincidences and excess items. Combined filtering tests for each pair (*A, B*) ∈ 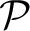 × 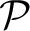 both the null hypotheses 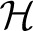
^*B*|*A*^_0_ (i.e., that *B* is a chance subset of *A*) *and* 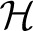
^*A*|*B*^_0_ (i.e., that *A* is a chance superset of *B*). If one of the null hypotheses is rejected, the corresponding pattern is retained as significant. Thus, if both hypotheses are rejected, both patterns are retained (*inclusive*). Accepting one null hypothesis does not necessarily lead to the rejection of the corresponding pattern (in contrast to subset or superset filtering): the pattern is rejected only if the other pattern is accepted, i.e., if the other null hypothesis is rejected. If both 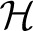
^*B*|*A*^_0_ and 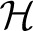
^*A*|*B*^_0_ are accepted, one of the two patterns is kept based on the covered-spikes criterion.

## 4. Calibration on artificial data

In this section we compare the performance (in terms of FPs and FNs) of PSF to PSF followed by PSR to illustrate the advantages yielded by the latter. For the sake of computational efficiency we employ the approximate versions of the tests for the subset and superset filtering with parameters *h* = 1 and *k* = 2, respectively. We test different types of artificial data that involve typical features of experimental data. After studying the general behavior of the analysis method for stationary, homogeneous data, we study data sets with heterogeneous firing rates across neurons, and with non-stationary firing rates in time.

### 4.1. Correlated data

As a model for data containing assembly activity, we generate correlated spike trains by a modified version of the single-interaction-process [SIP; Kuhn et al. (2003); Berger et al. (2010)], which we keep calling SIP for convenience. First, we simulate *N* = 100 parallel independent Poisson spike trains as background activity. Then we model assembly activity by inserting synchronous spike events in a subset of *z* of the *N* neurons (the *SIP neurons*, with ids 1 to *z*). This is done by generating a hidden Poisson process with the desired number *c* of pattern occurrences, from which spikes are copied into each of the *z* spike trains of the SIP neurons. Thus, as compared to the model proposed by Kuhn et al. (2003) we insert correlated firing only in a specific subset of the parallel processes. Before insertion of the synchronous patterns, the background firing rate of the SIP neurons is reduced by the rate of the hidden process to ensure the same firing rate for all neurons. In the simplest scenario, the firing rates and the pattern occurrence rate are stationary over time and homogeneous across neurons. More complicated cases will include either non-stationarity or heterogeneity of rates. The purpose of the analysis of such data is to test under controlled conditions if the simulated assembly is indeed detected and can be distinguished from background activity.

### 4.2. Independent data

To implement the null-hypothesis 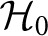 of complete independence needed to derive the significance of signatures of the correlated data, we generate independent Poisson processes of the same rates as the data to be tested, thus keeping the same marginal statistics. This is one way of implementing the null-hypothesis. However, in the context of analyzing real experimental data, one may want to keep more statistical features of the experimental data (e.g., non-stationary and heterogenous firing rates, deviation from Poisson, and so on). This can be realized by the use of more complex surrogates derived by manipulation of the original data, e.g., spike dithering (Grün, 2009; Louis et al., 2010b).

### 4.3. Assessing significance

We evaluate the performance of our analysis in terms of the average number of FPs and FNs obtained with PSF and PSR in *R* = 1000 iterations on the same model of correlated data (SIP of size *z* in *N* = 100 parallel spike trains). To study the performance of our analysis, we investigate 243 models differing in the size of the injected assembly *z* = 2, …, 10, its injection count *c* = 2, …, 10, and the firing rates *r* = 5, 10 or 20 Hz (here: homogeneous for all neurons). We analyse each model with a bin width *w* = 3 ms and *w* = 5 ms for the detection of synchronous spike patterns. See Table 1 for an overview of the parameter combinations. For the significance estimation we generate surrogate data, i.e., independent Poisson processes with the same firing rates as the correlated data, and analyse them with FIM as done for the correlated data. This procedure is repeated for *K* = 5000 times to derive the *p*-value spectrum and then the significance spectrum by employing an overall significance level of α = 0.01, Bonferroni-corrected for the number of signatures tested. The latter is given by the number of signatures existent in the correlated data, which never exceeded *m* = 50. In order to have the same corrected significance level for each of the 1000 iterations of each SIP model, we always correct for *m* = 50 tests, instead of correcting for the individual number *m*′ < *m* of signatures found in each data set. This yields the corrected significance level α_*_ = 2·10^−4^, which is typically more conservative than correcting individually for *m*′ tests. This procedure allows us to use a single significance spectrum for all 81 SIP models with the same firing rates, differing by parameters *z* and *c* only, and for all 1000 realizations of each model. To obtain the *p*-values with precision α_*_ we generate *K* = 1/α_*_ = 5.000 surrogates, compute their binary spectra and average them to draw the *p*-value spectrum (see Section 2.2).

**Table 1:** **Parameters for calibration of the method**.

**Simulation parameters**		**Analysis parameters**	
**Background activity**	**SIP**	**FIM**	**Statistical tests**
*N* = 100	*z* = 2, …, 10	*w* = 3,5 ms	α_*_ = 2·10^−4^
*r* = 5,10,20 Hz	*c* = 2, …, 10	*c*_0_ = 2	*K* = 5000
*T* = 3 sec		*z*_0_ = 2	*R* = 1000

Parameters for the background activity: N: number of neurons, r: firing rate, T: simulation time. Parameters for correlated data: z: number of neurons in correlated activity (size of SIP), c: SIP occurrences. Analysis parameters: w: bin width, c_0_: minimum item set support, z_0_: minimum item set size. Statistical parameters: α_*_: Bonferroni-corrected significance level (for m = 50 tests), K: number of surrogates, R: number of simulation runs per SIP model.

**Figure 4:**
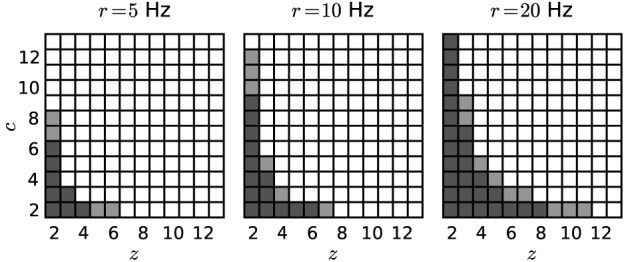
**Significance spectra for different parameter sets.** Independent Poisson spike trains (*N* = 100; *T* = 3 s) of different firing rates (*r* = 5, 10 or 20 Hz) serve as surrogates for the computation of three significance spectra (from left to right). Each square represents a (*z, c*) signature. Dark-shaded gray squares mark non-significant signatures obtained with *w* = 3 ms. Light-shaded squares represent further non-significant signatures for *w* = 5 ms. White squares indicate significant signatures for both choices of the bin width. Other parameters: *z*_0_ = 2, *c*_0_ = 2, α_*_ = 2·10^−4^, *K* = 5000.

Figure 4 shows significance spectra obtained from surrogate data for models differing by the firing rate *r* (5, 10 or 20 Hz) analysed with different bin widths *w* (dark gray for *w* = 3 ms, light gray for *w* = 5 ms; α_*_ = 2·10^−4^). The set of non-significant signatures shows a hyperbolic shape, which grows with both *r* and *w* to higher *z* and higher *c*. Both factors, higher firing rates and larger bin width, cause more spikes per bin, and therefore larger and more frequent chance patterns.

### 4.4. Performance, homogeneous firing rates

For each SIP parameter set we simulate the corresponding model *R* = 1000 times, and evaluate FPs and FNs of each realization. Their averages measure the performance of the analysis for each parameter constellation.

**Figure 5:**
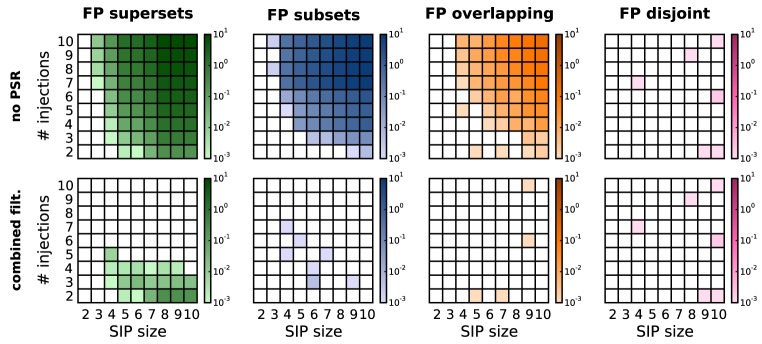
**Average number of FPs, distinguished by type, after PSF and PSR.** Average number of FPs obtained for different SIP models on *R* = 1000 model simulations. FPs are shown after performing PSF (top) and then PSR with combined filtering (bottom), and are distinguished by type (columns from left to right: FP supersets, FP subsets, FP overlapping, FP disjoint patterns). Each panel shows the average number of FPs obtained for different SIP models, each corresponding to a square in the grid: the models differ by the SIP size (from 2 to 10; *x*-axis) and its injection count (from 2 to 10; *y*-axis). Other parameters (same for all simulations): *N* = 100, *T* = 3 s, *r* = 20 Hz, *w* = 3 ms, *K* = 5000, α_*_ = 2·10^−4^.

As previously discussed (Section 3), in the presence of correlations PSF tends to classify chance subsets, supersets or overlapping sets as significant, thus yielding FPs. Figure 5, top row, shows this effect on simulations of SIP models differing by SIP size (*x*-axis of each panel) and injection count (*y*-axis). For each model, the FP level is computed as an average over 1000 stochastic simulations. The total amount of FPs increases as the SIP size and/or the number of injections get larger. The contribution of FP supersets (green) and FP subsets (blue) is about the same, while in comparison FP overlapping sets (yellow) occur only at higher values for *z* and *c*, and FP disjoint patterns (purple) are almost never observed. As shown in Figure 5, bottom row, PSR (here, combined filtering) largely reduces the amount of FPs. Although the PSR statistical tests apply to chance subsets (blue) and supersets (green) only (Section 3.2), they successfully remove most of the overlapping patterns (yellow) as well. The reason is that, if there is a CFIS *D* overlapping with the actual assembly *A* by *z*_0_ or more items, their intersection *B* is a CFIS as well (Figure 3C). In most cases PSF classifies *B* as significant together with *A* and *D*. If so, PSR likely rejects *D* when testing *H*^*D*|*B*^_0_, and rejects *B* when testing *H*^*B*|*A*^_0_.

**Figure 6:**
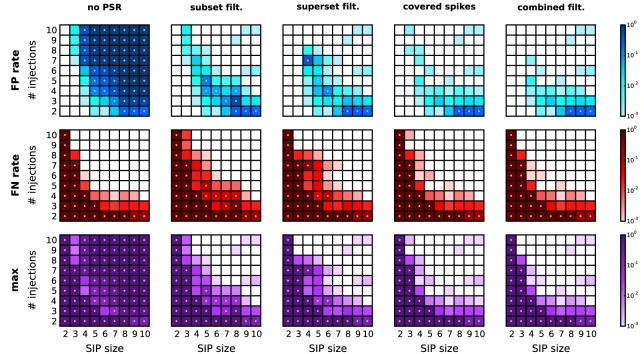
**Performance of PSR with homogeneous, stationary firing rates.** Performance of PSR with different filtering methods, measured as the fraction of *R* = 1000 simulations where FPs (top row) and FNs (second row) are detected (thus the fraction represents a rate). The maximum of the two (third row) indicates the combined error rate. Each matrix shows the performance for 81 different SIP models varying by SIP size (from 2 to 10, *x*-axis) and number of SIP injections (from 2 to 10, *y*-axis), of stationary and homogeneous neuronal firing rates (*r* = 20 Hz). The performance value is color-coded (see color bar, logarithmic scale). White squares mark SIP models where no simulations led to false outcomes. Gray dots mark entries where the error rate is above 5%. Each column corresponds to a different PSR strategy applied after PSF, from left to right: no filtering, subset filtering, superset filtering, covered-spikes criterion, combined filtering. Other parameters (same for all panels): *N* = 100, *T* = 3 s, *w* = 3 ms, *K* = 5.000, α_*_ = 2·10^−4^.

A reduction of the amount of FPs typically comes at the expense of enhanced FNs. In particular, FNs may occur if the real pattern is rejected in favor of one of its subsets or supersets. Figure 6 shows, for a range of combinations of SIP size and injection count, the resulting level of FPs, FNs, and the maximum of the two (as a measure of overall performance) after performing each of the proposed PSR strategies. The significance spectrum used to determine significance for all realizations of the SIP models is the one for *w* = 3 ms shown in Figure 4 (top right, dark-shaded entries). For the FPs shown in Figure 6, top row, the color-coded level refers to the fraction of simulations (out of 1000) containing one or more FPs. This measure takes values between 0 and 1, unlike the average FP counts shown in Figure 5. This representation simplifies the comparison with the average FN level, which ranges between 0 to 1 since here only a single spike pattern is injected in every simulation. To aid the comparison between the performances of PSF and PSR, gray dots mark those squares that correspond to models where the error rates exceed 5%. PSF on its own never performs well in terms of FNs and FPs simultaneously, while all PSR strategies yield a range of models for which both quantities are low. In summary, the relative improvement of PSR versus PSF shows that any PSR strategy reduces the FP rate considerably, while causing only a minor increase in the FN rate.

### 4.5. Performance, heterogeneous firing rates

If neurons have the same spiking statistics, the spike pattern statistics depends on the pattern size only. Thus, the *p*-value of each pattern is fully determined by the pattern signature. This does not hold when neurons have different spiking statistics, and in particular different firing rates. Here we discuss the case of heterogeneous firing rates across neurons, which are often present in electrophysiological data. Higher firing rates lead to a higher spiking probability per time bin. Patterns composed of neurons with higher firing rate are more likely to occur by chance, and are therefore less significant than patterns composed of neurons with lower rates. Thus, the *p*-values of patterns with the same signature (*z, c*) differ for different compositions of the firing rates. Pooling patterns by size and support in the pattern spectrum does not take into account the heterogeneity of firing rates across neurons and thus may lead to a biased statistics.

To investigate the robustness of our method against firing rate heterogeneity, we first simulate independent data consisting of 100 neurons, with a small population of neurons (2 to 10) firing at a higher rate (20 Hz) than the rest of the neurons (5 Hz). We simulate 1000 data sets of this type, and evaluate FPs in each of them by means of FIM and PSF (*K* = 5000 surrogates). In none of the simulations we detect significant signatures, i.e., FPs. The opposite scenario, where 2 to 10 neurons fire at 5 Hz and the others at 20 Hz, does not yield FPs as well. Thus, employing rate-preserving surrogates allows PSF to correctly estimate the significance of signatures under 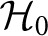, also when rates are heterogeneous across neurons.

**Figure 7:**
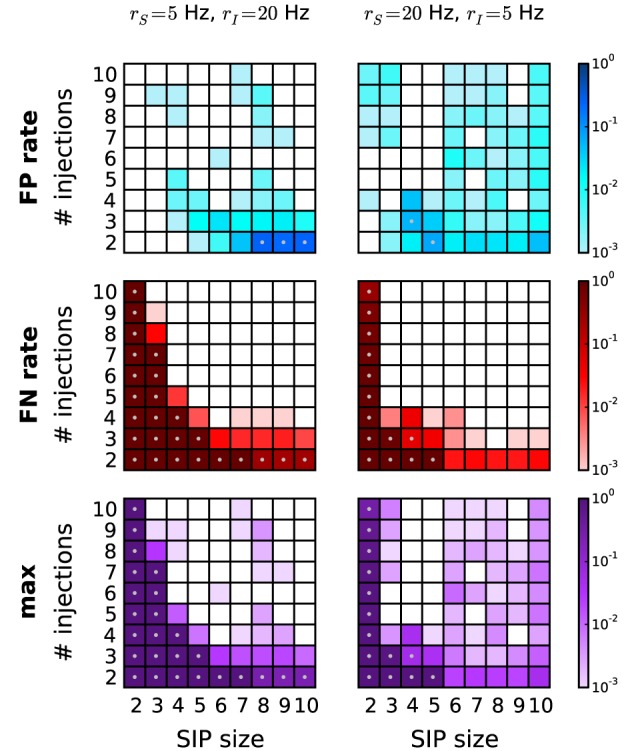
**Performance of PSR with heterogeneous firing rates.** Performance of PSR (combined filtering with parameters *h* = 1, *k* = 2) in terms of FP rates (*top* row), FN rates (middle row) and the combined error rates (maximum of FP and FN rates) (*bottom* row) of data with heterogeneous rates. Left column: SIP neurons fire at *r*_*S*_ = 5 Hz, independent neurons fire at *r*_*I*_ = 20 Hz. Gray dots mark entries where the error rate is above 5%. Right column: SIP neurons fire at *r*_*S*_ = 20 Hz and independent neurons at *r*_*I*_ = 5 Hz. Other parameters (same for all panels): *N* = 100, *T* = 3 s, *w* = 3 ms, *K* = 5.000, α_*_ = 2·10^−4^.

Next we study correlated data characterized by heterogeneous background firing rates. We investigate two cases based on a SIP model. In scenario S1, a pattern is injected in a set of neurons firing with lower firing rate (*r*_*S*_ = 5 Hz) than the independent neurons firing at rate *r*_*I*_ = 20 Hz (Figure 7, left column). In contrast, in scenario S2 the pattern is injected in neurons with higher firing rates (*r*_*S*_ = 20 Hz, *r*_*I*_ = 5 Hz; Figure 7, right column). In comparison to the homogeneous case where all neurons fire at 5 Hz (data not shown), the overall performance drops significantly, but does not so compared to the 20 Hz homogeneous case (see Figure 6, right column). This is consistent with the previous finding that higher rates worsen the performance by shifting the detection border in the significance spectrum to the right (Figure 4, left vs. right). This also explains why FP and FN rates in scenario S1 are higher than in scenario S2: the average firing rate in the former ranges (depending on the SIP model) from 18.5 to 19.7 Hz, in the latter from 5.3 to 7 Hz. Our choice of using PSR with combined filtering leads to a better performance in this scenario than the covered spikes criterion (not shown). Taken together, these results indicate that the method can deal well with heterogeneity of firing rates without severe performance loss.

### 4.6. Performance, non-stationary firing rates

**Figure 8:**
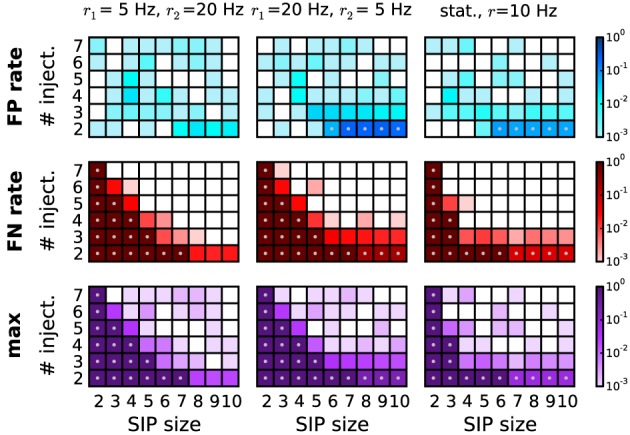
**Performance of PSR with non-stationary firing rates.** Performance of PSR (combined filtering) in terms of FP rate (top row), FN rate (middle row), and maximum of the two (bottom row), computed over *R* = 1000 simulations per SIP model. Each panel shows the performance for 54 different models varying by SIP size (from 2 to 10, *x*-axis) and SIP injections (from 2 to 7, *y*-axis). In the first two columns, the simulations consist of two epochs. The first epoch of duration *T*_1_ = 1.5 s is composed of a stationary, homogeneous SIP model (with firing rate *r* = *r*_1_), followed by a second epoch of *T*_2_ = 1.5 s with independent spiking at a rate of *r*_2_ ≠ *r*_1_. In the first column, the rate compositions are *r*_1_ = 5 Hz and *r*_2_ = 20 Hz, and in the second column *r*_1_ = 20 Hz and *r*_2_ = 5 Hz. For comparison, the third column shows the performance for the stationary case with all neurons firing at rate *r* = 10 Hz, and a duration of *T* = 3 s. Other parameters (same for all panels): *N* = 100, *w* = 3 ms, *K* = 5.000, α_*_ = 2·10^−4^. Gray dots mark entries where the error rate is above 5%.

Now we want to consider the case when the firing rates of the neurons are not stationary in time. To explore the sensitivity of our method to non-stationarities we employ simulated data, again consisting of 100 parallel spike trains, which fire in two consecutive epochs of length *T*_1_ and *T*_2_ (the total simulation time *T* = *T*_1_ + *T*_2_ is 3 s, as in the data previously analysed) at different rates (*r*_1_ = 5 Hz and *r*_2_ = 20 Hz; or vice versa), homogeneously across the neurons in both epochs. In the first epoch, correlated activity is inserted by the SIP model. SIP of size 2 to 10, injected 2 to 7 times, amount to a coincidence rate of 1.33 to 4.66 Hz in the first epoch. The background rate is reduced correspondingly. For comparison, we also study the stationary case, where all neurons fire at *r* = 10 Hz. The performance for the three scenarios is shown in Figure 8 (first column: *r*_1_ = 5 Hz, *r*_2_ = 20 Hz; second column: *r*_1_ = 20 Hz, *r*_2_ = 5 Hz; third column: *r*_1,2_ = 10 Hz). Although our analysis performs better (detection border more to the left) in the stationary case (*r* = 10 Hz; third column), it can still recover SIP activity with no FPs in a large portion of the parameter space, provided that rate-preserving surrogates are employed. As in the heterogeneous case, FPs increase when the SIP neurons have higher firing rates and thus more FP subsets occur. As apparent from Figure 8, bottom row, the method can correctly detect significant patterns in a wide range of models also in the presence of non-stationary rates. To study whether short transients in the firing rates tend to generate FPs, we repeated the analysis for *T*_1_ = 0.5 s, *T*_2_ = 2.5 s, setting first *r*_1_ = 5 Hz, *r*_2_ = 20 Hz and then *r*_1_ = 20 Hz, *r*_2_ = 5 Hz. In all cases we do not find enhanced FPs (data not shown), indicating that employing rate-preserving surrogates suffices to correct for rate non-stationarity in independent data.

## 5. Discussion

In this study we have presented a method to detect significant patterns of synchronous spiking in a subset of massively parallel spike trains in the presence of background activity. Our work is rooted in Picado-Muiño et al. (2013), where we demonstrated how to efficiently detect spike patterns in such data, and assess their significance under the null hypothesis of independent firing. Here we refined this significance test, which evaluates the significance of patterns using PSF on the basis of the pattern signature (size and support). PSF is prone to report FP patterns that arise due to the activation of an actual assembly mixed with chance synchrony because of background activity. To identify and remove these FP detections, we introduced here PSR as an additional statistical testing step. As shown in Figure 6 (second to last columns), PSR succeeds in eliminating FPs for a wide range of parameters, at the expense of a minor increase in FNs. A series of calibrations demonstrates the effectiveness of our approach under conditions of heterogeneous and non-stationary firing rates.

The relevance of higher-order correlations for information processing in the nervous system is hotly debated. Approaches based on maximum entropy models, such as Schneidman et al. (2006), suggest that higher-oder correlations contribute by a negligible fraction to the total network correlation, which appears to be dominated by pairwise correlations. However, it is important to stress that for correlations of a specific order, maximum entropy models estimate the overall magnitude of that correlation order, and are not sensitive to individual correlation structures of that order. Thus, the presence of a single group of correlated neurons with a certain size in the data is not enough for maximum entropy models to report significant correlation of the corresponding order. The study by Shlens et al. (2006) addresses this point, discussing that maximum entropy models may miss higher-order correlations because they overall contribute only by a negligible fraction to the total correlation. Besides, Roudi et al. (2009) showed that the statistical power of maximum entropy models describing spike correlations in heavily undersampled biological systems (such as parallel recordings with electrode arrays) is low. Despite these challenges, Ohiorhenuan et al. (2010) have shown using a maximum entropy model approach that in visual cortex local microcircuits exhibit evidence of higher-order interactions, whereas correlation statistics across long-range connections are explained on the basis of pair-wise interactions. However, methods designed to investigate individual spike patterns are needed to investigate the detailed structure of correlation in groups of spiking neurons.

A majority of current methods for spike correlation analysis limit themselves to fully synchronous patterns or to patterns with a specific size of typically low order [e.g., Grün et al., 2002a,b; Berger et al., 2007; Berger et al., 2010; Shimazaki et al., 2012]. Other approaches, such as CuBIC (Staude et al., 2010b), conclude on the presence of higher order correlations based on the statistics of the population activity without identifying the specific units engaged in such correlations. While Gansel and Singer (2012) presented a method for the detection of higher-order patterns, they identify pattern subsets by a purely heuristic procedure that is not accessible by analytic treatment, and that tests patterns directly, which requires a number of statistical corrections to avoid FPs (at the expense of FNs). Our proposed method instead first tests the significance of pattern signatures. PSF eliminates non-significant signatures based on surrogate data through the significance spectrum (see Figure 4), and determines the class 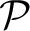 of associated significant patterns. Testing patterns on the basis of their signature rather than testing individual patterns reduces the number of required statistical tests to the number of signatures found in the data. We have shown that the composition of assembly and background spikes typically leads to the identification of additional significant patterns (i.e., FPs). In order to remove this type of FPs, we introduced here the PSR procedure that is based on conditional pairwise tests.

We have tested the performance of our analysis on artificial data where we embedded groups of synchronously spiking neurons in background activity of independent Poisson spike trains [SIP, cf. Kuhn et al. (2003)]. We studied the rate of FP and FN detections for occurrence rates of the synchronous pattern varying from 0.66 to 3.33 Hz, which reflect plausible values for the activation frequency of the assumed assemblies (Grün et al., 1999; Denker et al., 2010). The analysis shows in particular that by introducing PSR, assembly detection becomes possible with near perfect reliability and precision for a large range of SIP parameters. The transition shifts toward higher support and assembly size as the bin width or the firing rates increase (cf., Figure 4). Nevertheless, for physiologically realistic parameters only for very small or very infrequent SIP injections these patterns cannot be distinguished from chance synchrony. Moreover, evaluating patterns obtained from a larger set of simultaneously recorded neurons will have only minor impact on our findings due to a slight increase in the average size of observed patterns.

Non-stationarities of the firing rate in time or across neurons are a common concern faced by correlation analysis methods. The effect of non-stationary firing rates on PSF is two-fold. First, the surrogates used to calculate the significance estimates on pattern signatures should adequately reproduce the experimental rate profiles. Even if the underlying rate profile is not known, a variety of suitable approaches for surrogate generation is available for this task (Grün, 2009; Louis et al., 2010b). However, the sensitivity of detecting assembly activations is further affected by where these occur with respect to the rate non-stationarity. In this respect we tested the performance of PSF and PSR in a scenario of step-wise non-stationary firing rates where spike patterns were injected at selected rate levels only. Compared to the stationary case, the method retains a high performance for large parameter regimes (Figure 8), and shows only a slight increase in the number of FNs. For very large rate non-stationarities, a time-resolved analysis may be used to additionally aid the detection, as done, e.g., in the Unitary Events analysis (Grün et al., 2002b). In a similar framework, we found that also heterogeneous firing rates across neurons (Figure 7) exhibit a performance similar to the stationary case. While we see minor increases in the number of FPs, we remark that to a large extent these are indeed supersets of the injected pattern due to the high probability of gaining an additional coincident spike by chance from the set of neurons spiking at high rates.

**Figure 9:**
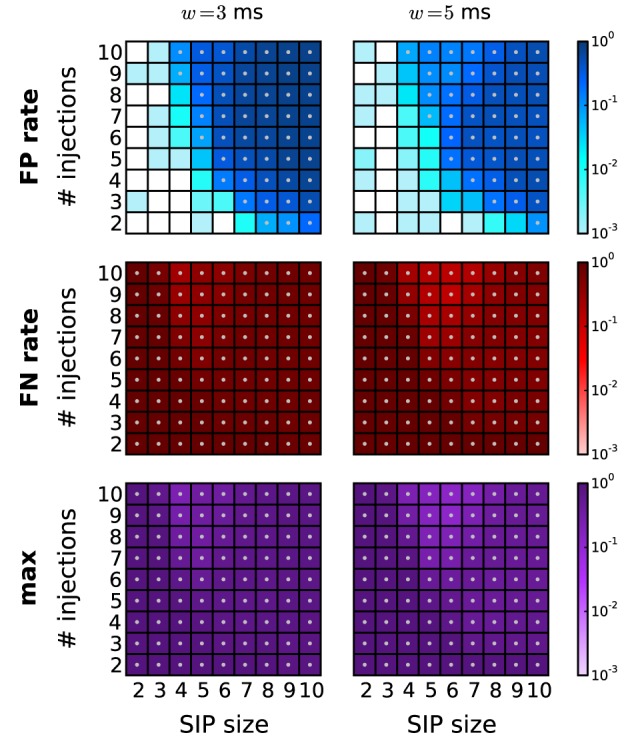
**Performance of PSR under jittered synchrony.** Performance of PSR (combined filtering with *h* = 1, *k* = 2) on data from SIP models with jittered synchrony. The spikes of SIP events are randomly jittered up to ±1 ms around the original occurrence time. The performance is shown in terms of FP rates (first row), FN rates (middle row) and maximum of the two (bottom row) for different bin widths: *w* = 3 ms (left column) and *w* = 5 ms (right column).

In this study we assumed that assemblies occur at the time resolution of the data, i.e., that spike times of the assemblies are not jittered in time. In electrophysiological data this is a rare scenario, and instead spike synchrony typically occurs with a temporal jitter of up to several milliseconds [Grün et al., 1999; Pazienti et al., 2008]. In order to capture such slightly imprecise synchrony, exclusive binning is typically applied (Grün et al., 1999), where the bin width is chosen large enough to capture the jittered spike pattern. However, the spikes of the pattern may be split into adjacent bins with a probability that depends on the jitter, bin size, and pattern size. Therefore, the original synchronous events are destroyed, leading to increased FN rates (Grün et al., 1999). In Figure 9 we show how this effect can have a substantial impact on the performance of the method. We applied PSF followed by PSR (combined filtering) on data where synchronous patterns are injected with a jitter of ±1 ms, and analysed with a bin width of *w* = 3 ms (left column) and *w* = 5 ms (right column). The performance drops considerably due to an increase of the FP rate for higher *z* and *c*, and an overall increase of the FN rate. The performance is slightly better for a bin width of 5 ms. Consistent with these findings, Grün et al. (1999) showed that for two parallel spike trains about 60% of the synchronous events are lost if the bin width corresponds to the jitter width. An earlier modification of exclusive time binning [multiple shift method, Grün et al., 1999] that avoids the splitting of jittered synchrony was not trivially applicable to large numbers of parallel spike trains. In Picado-Muiño et al. (submitted) we demonstrate how to implement a method for pattern detection based on the inter-spike distances rather than discrete time binning. This approach successfully detects jittered spike patterns and therefore trivially exhibits a performance in the context of PSF that is similar to that achieved in the absence of jitter (see Picado-Muiño et al., submitted, for details). Thus, it also complements the PSR framework presented in this study. Therefore, we suggest to detect jittered synchrony by the continuous detection method and perform the analysis by the proposed sequence of FIM, PSF, and PSR.

A further scenario that remains to be addressed in the future is unreliability in spiking activity that causes neurons to selectively skip participation in assembly activations. This scenario was discussed in the context of the synfire chain model, where it was shown that stable propagation of synchronous spike packages through the network happens reliably although the probability that individual neurons participate in each activation of the synfire chain is lower than 1 (Diesmann et al., 1999). Selective participation may arise as a consequence of synaptic failure. The multiple interaction process [MIP; Kuhn et al., 2003] was proposed as a stochastic model implementing such a behavior. Our method would interpret the variable composition of spikes in a single MIP event as occurrences of multiple SIP events of lower support.

We conclude with a discussion of the practical implementation of the proposed analysis on data from electrophysiological recordings. Given a set of parallel spike recordings obtained at a resolution (i.e., binning) *w*, we choose the minimum pattern size *z*_0_ and the minimum pattern support *c*_0_ of the analysis. First, the spike data is binned and, using FIM, the CFISs and the corresponding pattern signatures are obtained from the transaction list. While this approach is feasible for the experimental data available today, with several hundreds of parallel recordings the computational effort may become too large. In this scenario, we suggest to pre-filter the data entering the analysis as suggested by Berger et al. (2010) before applying FIM on the reduced set of neurons. To monitor dynamic changes in the correlation structure of the activity, e.g., if assemblies are time locked to a particular behavioral event, one may choose to additionally perform the analysis in sliding windows.

Next, the significance of the observed patterns is evaluated by PSF under the null-hypothesis of full independence implemented by uncorrelated surrogate data. For experimental data, several techniques for surrogate generation based on stochastic sampling have been proposed in the past [for a review, see Grün, 2009]. Surrogates that preserve the firing rate profiles, such as spike dithering, seem most appropriate since PSF determines pattern significance based on the firing rates. Given the significance level α and *m* detected pattern signatures, a minimum of *K* = ⌈*m*/α⌉ surrogates are required to achieve the Bonferroni-corrected significance level α_*_ = α/*m*. Once the surrogates have been generated, we follow the procedure described for the simulated data. CFISs, pattern signatures and the resulting binary pattern spectrum are obtained for each surrogate run. Next, the *p*-value spectrum is obtained as an average of the binary spectra (see Section 2.2). The signatures whose *p*-values do not exceed the Bonferroni-corrected significance level α_*_ are marked as significant, and the CFISs of significant signatures are collected into the class 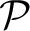 of potential assemblies. Finally, PSR with combined filtering is performed to reduce 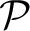 to a subclass 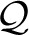 of patterns which are mutually significant with respect to each other.

In summary, the use of FIM combined with the statistical tests described in this study and in Picado-Muiño et al. (submitted) represents a powerful tool to extract candidate assemblies from experimental data. The method is statistically rigid, computationally feasible, robust against heterogeneity in the data, and powerful enough to deal with the limited amount of data typically available from electrophysiological experiments. We expect that our approach will help to reveal how precise spike synchronization observed by pairwise analysis in relation to behavior (Riehle et al., 1997) is manifested at the level of neuronal populations.

### Conflict of interest statement

The authors declare that the research was conducted in the absence of any commercial or financial relationships that could be construed as a potential conflict of interest.

## Software and supplemental material

The FIM library underlying the Python scripts with which we carried out our experiments is available at http://www.borgelt.net/pyfim.html. Python and shell scripts for related experiments as well as more extensive result diagrams are available at http://www.borgelt.net/accfim.html and http://www.borgelt.net/cocofim.html. Please also consult http://www.spiketrain-analysis.org for these codes and further information on the analysis of parallel spike trains.
